# Lipoxygenase Activity Accelerates Programmed Spore Germination in *Aspergillus fumigatus*

**DOI:** 10.3389/fmicb.2017.00831

**Published:** 2017-05-09

**Authors:** Gregory J. Fischer, William Bacon, Jun Yang, Jonathan M. Palmer, Taylor Dagenais, Bruce D. Hammock, Nancy P. Keller

**Affiliations:** ^1^Department of Genetics, University of Wisconsin–Madison, MadisonWI, USA; ^2^Department of Medical Microbiology and Immunology, University of Wisconsin–Madison, MadisonWI, USA; ^3^Department of Entomology and Nematology and Comprehensive Cancer Center, University of California, Davis, DavisCA, USA

**Keywords:** *Aspergillus fumigatus*, spore germination, arachidonic acid, lipoxygenase activity, linoleic acid

## Abstract

The opportunistic human pathogen *Aspergillus fumigatus* initiates invasive growth through a programmed germination process that progresses from dormant spore to swollen spore (SS) to germling (GL) and ultimately invasive hyphal growth. We find a lipoxygenase with considerable homology to human Alox5 and Alox15, LoxB, that impacts the transitions of programmed spore germination. Overexpression of *loxB* (*OE::loxB*) increases germination with rapid advance to the GL stage. However, deletion of *loxB* (*ΔloxB*) or its signal peptide only delays progression to the SS stage in the presence of arachidonic acid (AA); no delay is observed in minimal media. This delay is remediated by the addition of the oxygenated AA oxylipin 5-hydroxyeicosatetraenoic acid (5-HETE) that is a product of human Alox5. We propose that *A. fumigatus* acquisition of LoxB (found in few fungi) enhances germination rates in polyunsaturated fatty acid-rich environments.

## Introduction

Pathogenic and saprophytic fungi begin interactions with their environment through contact of airborne conidia with a host or substrate. In response to the right signals, this can lead to spore germination followed by extensive hyphal (vegetative) growth. The genus *Aspergillus*, composed of *ca.* 300 species, contains both pathogenic and saprophytic species which break spore dormancy through three distinct stages of spore maturation, collectively known as the germination process. The dormant spore (DS), or resting spore/conidium, when activated by specific cues undergoes isotrophic growth and swells to almost double its initial size into what is termed the swollen spore (SS). Additional cues signal polarized germ tube emergence from the SS to form a germling (GL) which may or may not lead to extensive hyphal growth.

Genetic approaches to identify phase-specific germination mutants have identified only a handful of candidates. A genetic screen for temperature-sensitive germination mutants of *A. nidulans* identified genes directly involved in translation and protein folding, a putative malonyl-CoA synthatase, and a *ras* homolog (Osherov and May, 2000). Overexpression of dominant negative forms of *rasA* in *A. nidulans* delays germination, whereas overexpression of dominant active forms yields large multinucleate spores where early germ tube formation is inhibited (Som and Kolaparthi, 1994). Spermidine levels are also important for progression from GL to hyphal growth, and deletion of the spermidine synthase gene *spdA* results in enlarged multinucleate spores that can germinate but not proceed to hyphal growth (Jin et al., 2002). Additionally, many studies have described delays in the general germination process in *Aspergillus* spp., either associated with genetic mutations or treatment with chemicals, but provide little detail on the effects on specific aspects of the germination program (Liebmann et al., 2004; Kwon et al., 2010; and reviewed in Osherov and May, 2001).

The environmental triggers which initiate germination of DSs are thought to include sugars, amino acids, and inorganic salts (Carlile and Watkinson, 1994). Studies with *Neurospora crassa* found a carbon source and salt are sufficient to initiate germination, but a carbon source alone is sufficient to induce germination in *A. nidulans* (Schmit and Brody, 1976; Osherov and May, 2000). Surface hydrophobicity and hardness regulates spore germination in *Magnaporthe grisea* (Talbot, 1995). Host compounds such as the isoflavanoid pisatin induce germination of *Fusarium solani* (Ruan, 1995). Alternatively, compounds produced by fungi themselves can inhibit germination. The auto-inhibitors *cis*-ferulic acid methyl ester and 3,4-dimethoxycinnamic acid methyl ester inhibit germination of certain rust fungi (Macko et al., 1971). Another auto-inhibitor, 1-octene-3-ol, is an oxylipin volatile derived from the oxygenation and breakdown of linoleic acid (LA). Density-dependent germination effects have been attributed to 1-octene-3-ol in *Penicillium paneum* (Chitarra et al., 2004) and *A. nidulans* (Fischer et al., 1999; Herrero-Garcia et al., 2011), although conflicting results have been observed in *A. flavus* (Miyamoto et al., 2014).

Oxylipins such as 1-octene-3-ol are derived from the incorporation of molecular oxygen into a diverse set of polyunsaturated fatty acids (PUFAs) by oxygenases, including lipoxygenases (LOX), cyclooxygenases (COX), and cytochrome P450 enzymes. The ability of oxylipins to influence biological responses in fungi, plants, and mammals has been well-described (for review, see Fischer and Keller, 2016). However, the actual effects of PUFAs from which oxylipins are derived on fungal development has lacked investigation.

There is considerable evidence that fatty acids have inherent antibacterial and antifungal properties, specifically against plant and human pathogenic fungi (Bergsson et al., 2001; Walters et al., 2003; Liu et al., 2008; Thibane et al., 2012) including *Aspergillus* species (Pohl et al., 2011). PUFAs have been documented to reduce biomass of the plant pathogens *Pyrenophora avenae* and *Crinipellis perniciosa* (Walters et al., 2004). Additionally, PUFAs including LA induce DNA fragmentation in *Candida albicans*, indicative of late stage apoptosis (Thibane et al., 2012). Despite the potential utility of PUFAs as antifungal agents, little is known about the mechanism(s) by which fungi may overcome their antimicrobial activity and specifically what aspect of development (mycelial or germination) is impeded. We set out to investigate whether one potential mechanism by which *A. fumigatus* could overcome this antimicrobial activity is through expression of fatty acid metabolizing enzymes such as LOX.

While mammals have several LOX, the enzyme class is relatively rare in fungi. Those fungal LOX already identified are classified in two groups based on their C terminal sequence: either a conserved WRYAK motif with a C-terminal isoleucine or a WL-L/F-AK motif with a C-terminal valine (see phylogentic analysis in Heshof et al., 2014). C-terminal valine lipoxygenase also contain signal peptides implicating them as secreted LOXs. Only a few *Aspergillus* spp. contain any *lox* genes in their genomes. *Aspergillus flavus* encodes one lipoxygenase, *Aflox*, (a valine-type Lox) with a role in quorum sensing and developmental switching from asexual to sexual growth (Horowitz Brown et al., 2008). Interestingly, the *A. fumigatus* genome contains two LOX, one Val-Lox (Afu4g02770, here on referred to as *loxB*) homologous to the sole *A. flavus* Lox, and one Ile-Lox (Afu7g00860, *loxA*). Purification and *in vitro* reaction found LoxB to contain manganese as a co-factor and predominantly synthesize 13-hydroperoxyoctadecadienoic acid, the unstable precursor to 13-hydroxyoctadecadienoic acid (13-HODE) derived from LA (Heshof et al., 2014).

Considering its ubiquitous distribution and the prevalence of *lox* genes within *A. fumigatus*, we investigated possible developmental effects from its activity on two PUFA substrates, LA and arachidonic acid (AA). As reported here, we find LoxB is an important mediator in the transition stages of programmed germination. Overexpression of *loxB* significantly increases the ability of *A. fumigatus* spores to progress to GL stage whereas deletion of *loxB* or its signal peptide region delays progression to the SS stage in the presence of the fatty acid, AA. However, this delay is remediated by the addition of 5-hydroxyeicosatetraenoic acid (5-HETE), an oxylipin normally derived from the oxygenation of AA by the mammalian lipoxygenase Alox5. We propose that the acquisition of LoxB may represent a mechanism by which *A. fumigatus* enhances germination rates in environments where particular fatty acids are prevalent, providing a competitive advantage for the fungus.

## Materials and Methods

### Fungal Strains and Culture Conditions

All strains utilized or developed are listed in **Table 1**. Strains were propagated on solid glucose minimal media (GMM), amended as necessary with supplements for auxotrophs, at 37°C (Shimizu and Keller, 2001). *A. fumigatus* asexual spores were collected in water supplemented with 0.01% Tween 80, enumerated using a hemocytometer and maintained as glycerol stocks. Spore suspensions of the various mutants were used to inoculate liquid GMM for 13-HODE quantification.

**Table 1:** *Aspergillus fumigatus* strains used in this study.

Fungal strain	Genotype	Source or reference
AF293	Wild type	Xue et al., 2004
AF293.1	*pyrG1*	Osherov et al., 2001
AF293.6	*pyrG1 argB1*	Xue et al., 2004
TGJF1.5	*pyrG1; gpdA(p):loxB A. parasiticus pyrG*	This study
TGJF1.7	*pyrG1; gpdA(p):loxB A. parasiticus pyrG*	This study
TTRD51	*pyrG1, argB1, ΔloxB::A. fumigatus argB*	This study
TJMP39.6	*pyrG1 argB1, ΔloxB::A. fumigatus argB, A. parasiticus pyrG*	This study
TGJF33.6	*pyrG1 argB1, ΔloxB::A. fumigatus argB, loxB(p):loxB A. parasiticus pyrG*	This study
TGJF34.9	*pyrG1, argB1, ΔloxB::A. fumigatus argB gpdA(p):loxB A. parasiticus pyrG*	This study
TGJF34.10	*pyrG1, argB1, ΔloxB::A. fumigatus argB gpdA(p):loxB A. parasiticus pyrG*	This study
TGJF35.4	*pyrG1, argB1, ΔloxB::A. fumigatus argB gpdA(p):Δ1-20:loxB A. parasiticus pyrG*	This study
TGJF35.5	*pyrG1, argB1, ΔloxB::A. fumigatus argB gpdA(p):Δ1-20:loxB A. parasiticus pyrG*	This study
TGJF36.1	*pyrG1, argB1, ΔloxB::A. fumigatus argB gpdA(p):Δ1-26:loxB A. parasiticus pyrG*	This study
TGJF36.3	*pyrG1, argB1, ΔloxB::A. fumigatus argB gpdA(p):Δ1-26:loxB A. parasiticus pyrG*	This study
TGJF43.8	*pyrG1, argB1, ΔloxB::A. fumigatus argB gpdA(p):gfp:loxB A. parasiticus pyrG*	This study
TGJF44.14	*pyrG1, argB1, ΔloxB::A. fumigatus argB gpdA(p):Δ1-20:gfp:loxB A. parasiticus pyrG*	This study
TGJF45.15	*pyrG1, argB1, ΔloxB::A. fumigatus argB gpdA(p):Δ1-26:gfp:loxB A. parasiticus pyrG*	This study

### Creation of Lipoxygenase Mutants

Construction, isolation, and maintenance of recombinant plasmids was performed according to standard methods (Sambrook and Russell, 2001). Primers used are listed in Supplementary Table S1. The ORF of *A. fumigatus loxB* (Afu4g02770) was identified via the AspGD database^1^.

Deletion of *loxB* was achieved by creating a double-joint PCR fragment consisting of approximately 1 kb flanking regions of *loxB* and utilization of the *argB* gene of *A. fumigatus*. Briefly, the regions upstream and downstream of *loxB* were amplified using “TDLoxB P1 F” with “TD LoxB P3 R” and “TD LoxB P4 F” with “TD LoxB P6 R,” respectively. The *A. fumigatus argB* gene was amplified using “JP Afumi argB F” and “JP Afumi argB R.” The corresponding *loxB*-disruption fragment was amplified using the three PCR fragments described above with the primer pair “TD LoxB P2 F” and “TD LoxB P5 R” and used to transform AF293.6 to arginine prototrophy according to previously published methods (Szewczyk et al., 2006). The strain TTRD51 was isolated and confirmed to be a single integration disruption mutant by Southern blot (Supplementary Figure S1A). TTRD51 was then transformed to prototrophy with *A. parasiticus pyrG* via the plasmid pJW24 (Calvo et al., 2004). Single copy integration of *A. parasiticus pyrG* was confirmed by Southern blot, which resulted in the prototrophic *ΔloxB* strain, TJMP39.6 (Supplementary Figure S1A).

An overexpression *loxB* plasmid construct, pGJF1.1, carrying *A. parasiticus pyrG* as a marker gene was developed as described. “GF gpdA/loxB-loxB(t) F” and “GF loxB(t) XbaI Site R” were used to amplify the *loxB* ORF and a 0.581 kb 3′ flanking region. These primers introduced 30 base pairs of the 3′ end of the *A. nidulans* glyceraldehyde-3-phosphate dehydrogenase (*gpdA)* promoter (which was used to overexpress *loxB*) to the 5′ end of the PCR product and an *XbaI* restriction site to the 3′ end of the PCR product. The *A. nidulans gpdA* promoter was amplified from pJMP8 (Sekonyela et al., 2013) using “GF gpdA F” and “GF gpdA/loxB R” which introduced a *SpeI* restriction site to the 5′ end of the product and 30 bp of the 5′ region of the *loxB* PCR product to the 3′ end of the *gpdA* amplicon. In order to fuse the *loxB* PCR product downstream of the *gpdA* promoter amplicon, fusion PCR was performed as previously described Szewczyk et al. (2006). The *gpdA*:*loxB* fusion product was digested with *XbaI* and *SpeI* and ligated into pJMP7 (Sekonyela et al., 2013), an *A. parasiticus pyrG*-containing plasmid. pGJF1.1 was used to transform AF293.1 to yield TGJF1.5 and TGJF1.7 and TTRD51 transformed to produce TGJF34.9 and TGJF34.10 (**Table 1**) (Szewczyk et al., 2006). Verification of ectopic plasmid integration was confirmed by PCR and Southern blot (Supplementary Figure S1A).

Complementation of *loxB* was achieved by amplifying the flanking intergenic region of *loxB* using the primer pair “GF loxB Complement F” and “GF loxB Complement R,” which introduced *NotI* and *SpeI* sites at the end of the *loxB* intergenic DNA fragment. The *loxB* cassette was digested with *NotI* and *SpeI* and ligated into pJMP7, yielding the plasmid pGJF22.1. This plasmid was then transformed into TTRD51, yielding the prototrophic ectopic *loxB* complement strain, TGJF33.6. Integration was confirmed via Southern blot (Supplementary Figure S1A).

### Development of *gfp-loxB* Fusion Construct and Signal Peptide Mutants

For deletion of the signal peptide of *loxB*, pGJF1.1 was subjected to quick-change mutagenesis using a modified approach as previously described Bok and Keller (2012). For *loxB* lacking the signal peptide region, “GF d1-20:loxB F” was used to amplify a modified version of pGJF1.1 lacking residues 1-20 (ΔSP1) yielding pGJF10.1, while “GF d1-26:loxB” was used to amplify a modified version yielding pGJF11.2 which lacked N-terminal residues 1-26 (ΔSP2).

Tagging of LoxB with *gfp* was achieved via modifications to pGJF1.1, pGJF10.1, and pGJF11.2 using quick-change mutagenesis. The primer pair “GF loxB-Nterm GFP F” and “GF loxB-Nterm GFP R” were used to amplify a *gfp-*GA linker amplicon from a synthesized G-Block fragment (IDT) with 30 bp of homology to sequence directly upstream and downstream of LoxB residues 30 and 31. This nested location was selected since it was downstream of the putative signal peptide regions but upstream of the predicted lipoxygenase domain. The G-block was synthesized using the sequence of *gfp* from pFNO3 (Yang et al., 2004) with the addition of a C-terminal 5x glycine/alanine linker codon optimized for *A. niger*. The purified *gfp* cassette was used to amplify gfp-tagged versions of full-length *loxB* (pGJF26.3), ΔSP1 *loxB* (pGJF27.2), and ΔSP2 *loxB* (pGJF28.5).

Together, pGJF10.1, pGJF11.2, pGJF26.3, pGJF27.2, and pGJF28.5 were all independently transformed into the fungal strain TTRD51, yielding TGJF35.4 and 35.5, TGJF36.1 and 36.3, TGJF43.8, TGJF44.14, and TGJF45.15, respectively. Integration of the corresponding plasmids was verified via Southern blot (Supplementary Figure S1A) based on genome structure outlined in Supplementary Figure S1C.

### Verification of Expression via Northern Blot and Semi-qPCR

Disruption and overexpression of *loxB* was confirmed using a combination of northern blots and semi-qPCR. For northern blotting, total RNA was extracted with TRIzol reagent from lyophilized mycelia. Probes for northern analysis are indicated in Supplementary Figure S1B and labeled with dCTP-αP^32^. Because transcript levels for *loxB* were not detectable in wild type nor the complementation strain (Supplementary Figure S1D), semi-qPCR was used to further verify wild type *loxB* expression, disruption, and complementation (Supplementary Figure S1E). Semi-qPCR was carried out as follows: 5 μg of RNA from lyophilized mycelium was treated with DNAseI for 1 h at 37°C. Five hundred nanogram of DNAse-treated RNA was converted to cDNA using the Bio-Rad (Hercules, CA, USA) iScript cDNA synthesis kit according to manufacturer’s protocol. Twenty five nanogram of cDNA was used as template for cDNA-specific amplicon production. For *loxB* cDNA, the primer pair “GF loxB qPCR F” and “GF loxB qPCR R” was used to amplify a 225 bp amplicon lacking *loxB* intron 2. For *loxA*, a cDNA amplicon was produced via PCR using the primer pair “GF loxA seq 1F” and “GF loxA Probe R.” Actin cDNA served as a loading control and an amplicon was produced via PCR using the primer pair “FY act1 RT FOR” and “FY act1 RT REV.”

### Fatty Acid Germination Assay

Germination assays were carried out on spores derived from the various *loxB* mutant strains. Two milliliters of 1 × 10^5^ spores/mL in GMM supplemented with 0.5% tergitol (Sigma, St. Louis, MO, USA) in the presence or absence of AA or LA (Sigma, St. Louis, MO, USA) were used to inoculate each well of a Costar^®^ 12-well dish (Corning, Corning, NY, USA). Microscopic images were captured using a Nikon Eclipse T*i* inverted microscope equipped with a OKO-Lab microscopic enclosure to maintain the temperature at 37°C for *A. fumigatus* (OKO Lab, Burlingame, CA, USA). Germinated spores were observed using a Nikon Plan Fluor 20xPh1 DLL objective and phase-contrast images captured every 1–2 h using the Nikon NIS Elements AR software package (v. 4.13). Germinated spores were counted with slight modifications as previously described Dagenais et al. (2008). Briefly, a DS was considered swollen if the diameter was double in size to DSs or a GL if an emerging germ tube was clearly present. One hundred spores were observed for each strain (*n* = 3) and growth condition. Values in figures represent the average percentage of spores germinated ± SEM. The Student *t*-test was carried out to determine statistical significance using the GraphPad Prism software (La Jolla, CA, USA).

Mycelial growth in the presence of AA was monitored using an OD_600_ reading in a 96-well plate format. To establish a mycelial network, 10^5^ spore were inoculated in GMM +0.5% tergitol and grown overnight at 37°C. Initial absorbance values were collected using a BioTek EPOCH 2 microplate reader with Gen5 acquisition software. AA was added to wells at a final concentration of 1.0, 0.5, and 0.25 μg/μL to challenge mycelial growth. Twenty-two hours after AA treatment, another absorbance reading was collected and the net growth calculated.

### Microscopy

Fluorescent microscopy of GFP localization was carried out on the same platform but using a 60x Nikon Plan Apo VC Oil DIC objective. Briefly 10^4^ spores were used to inoculate glass coverslips immersed in GMM + 0.5% tergitol overnight at 30°C (to reduce sample autofluorescence), after which the slides were imaged. Spores were inoculated directly on a coverslip and imaged similarly.

### Quantification of *loxB* via qRT-PCR

For quantification of *loxB* expression in the presence of AA and LA, quantitative RT-PCR was carried out as follows: RNA for cDNA synthesis was isolated as previously described for semi-qPCR from total fungal tissue after 29 h of growth (increase tissue yield) in the presence of 0.5 μg/μL LA and AAs. Samples were analyzed in a volume of 20 μL using iQ SYBRGreen Supermix (Bio-Rad Laboratories, Inc.). Reactions were performed in triplicate using cDNA template for *loxB* and actin. A mastermix of SYBRGreen and primers was prepared for each primer pair (*loxB*: “GF loxB qPCR F with “GF loxB qPCR R,” actin: “FY act1 RT FOR” with “FY act1 RT REV”). Each reaction contained 25 ng of cDNA and a final primer concentration of 300 nM. Reactions were performed with the MyiQ Real-Time PCR detection system (Bio-Rad Laboratories, Inc.) using the “2-step amplification plus melting curve” protocol: 95°C for 3 min followed by 40 cycles of 95°C for 1 min. (denaturation) and 55°C for 45 s (annealing and elongation). The determination of the threshold values (Ct) was generated automatically by the MyiQ software. The identities of the amplicons and the specificity of the reactions (absence of primer-dimers) were confirmed by the melt curve profile of the amplified products. The amount of *loxB* cDNA was standardized to the amount of the actin internal standard cDNA for each sample and normalized to *loxB* expression with no fatty acid treatment (vehicle) (*n* = 3). Differences in the adjusted mean values (*n* = 3 ± SEM) among the treatments were analyzed for statistical significance using students *T*-test via GraphPad Prism version 6.00 for Windows software.

### Quantification of 13-HODE

To confirm the functionality of our deletion and overexpression *loxB* strains, Costar^®^ 12-well cell culture plates (Corning, Corning, NY, USA) containing 3 mL of GMM media were inoculated with spores of wild type, *ΔloxB and OE::loxB A. fumigatus* mutants to a final concentration of 1 × 10^6^ spores/mL (*n* = 3). Plates were covered with AeraSeal^TM^ Breathable Sealing Film (Excel Scientific, Victorville, CA, USA) and incubated for 3 days at 37°C and 185 rpms. The antioxidant cocktail (0.2 mg/mL BHT, TPP, and EDTA) were added to prevent oxylipin degradation.

The culture supernatants were extracted using solid phase extraction protocol as described previously (Yang et al., 2009). The reconstituted solutions were injected onto a UPLC/MS/MS system (Agilent 1200 SL system, Santa Clara, CA, USA) coupled to AB Sciex 4000 Qtrap MS/MS system (Foster City, CA, USA). The detailed parameters were described in a previous paper (Zivkovic et al., 2012). Limit of quantification (LOQ) for 13-HODE is presented in Supplementary Table S2.

Levels of 13-HODE between wild type, *ΔloxB, and OE::loxB* strains were analyzed for significance using students *T*-test via GraphPad Prism version 6.00 for Windows, GraphPad Software. (La Jolla, CA, USA^2^). Values in figures are expressed as mean (*n* = 3) ± SEM.

## Results

### Identification of 5-Lox and 15-Lox Homologs Within *Aspergillus fumigatus*

**FIGURE 1:**
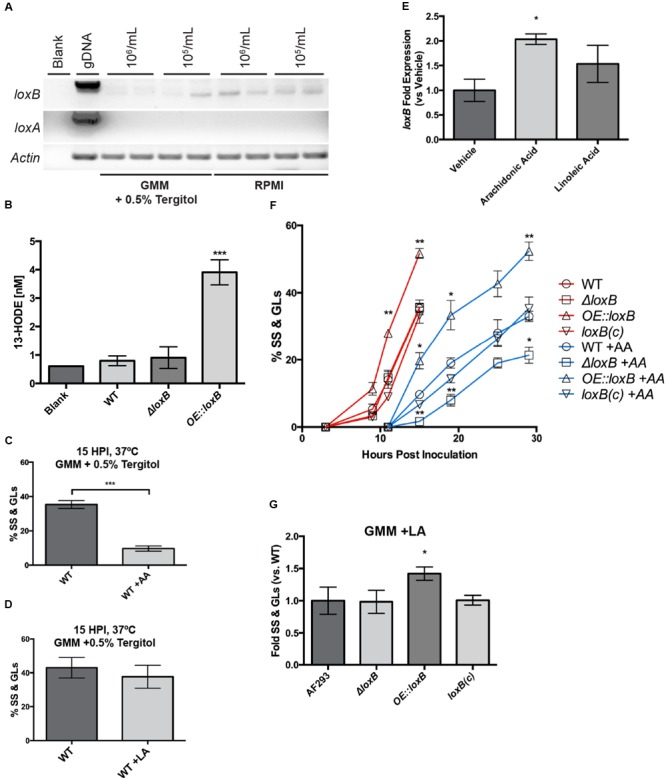
**LoxB is up-regulated upon arachidonic acid exposure and promotes germination. (A)** *loxB* but not *loxA* is expressed in glucose minimal media (GMM) +0.5% tergitol and RPMI. Different spore concentrations of 1 × 10^6^ and 1 × 10^5^ spores were tested to look at density-dependent effects on lipoxygenase expression after 48 h of growth at 37°C. *loxB* was up-regulated at 1 × 10^6^ spores/mL in RPMI media compared to GMM. Actin served as a loading control for semi-qPCR analysis. **(B)** Verification of LoxB enzymatic activity in novel fungal strains. The previously documented product of *A. fumigatus* LoxB (13-HODE) was assessed in WT, *ΔloxB*, and *OE::loxB* strains. While significant levels of 13-HODE were identified in *OE::loxB* supernatant, background levels equivalent to those identified in the blank were recorded in WT and *ΔloxB* supernatants. **(C)** Addition of 0.5 μg/μL arachidonic acid (AA) reduces the number of wild type swollen spores (SS) and germlings (GLs) compared to mock treatment, however, the same concentration of linoleic acid (LA) does not **(D)**. **(E)** *loxB* is up-regulated in the presence of AA after 29 h at 37°C. Values are average (*n* = 3) fold expression ± SEM after standardization to wild type tissue from mock treatment (vehicle). **(F)** Germination assay for *loxB* strains grown in GMM + 0.5% tergitol in the presence and absence of AA. Addition of AA significantly delays SS and GL formation of all strains compared to the mock treatment. **(G)** Upon exposure to 0.5 μg/μL LA, no difference in growth is observed after 15 hpi between wild type, *ΔloxB*, and *loxB(c)* complement strains. However, the increase in SS and GLs when *loxB* is overexpressed is maintained as in **(F)**, suggesting delayed germination of the *ΔloxB* strain is a function of AA treatment, whereas accelerated germination of the *OE::loxB* strain is not. Values represent average of *n* = 3 trials ± SEM and Student’s *t*-test was used to identify statistical differences, ^∗^*p* < 0.05, ^∗∗^*p* < 0.01, ^∗∗∗^*p* < 0.001.

The human Alox5 protein sequence was used to identify homologs within the *A. fumigatus* genome. Genome-wide BLAST analysis identified two LOX with considerable similarity: LoxA (24.2% identity, Afu7g00860) and LoxB (24.3% identity, Afu4g02770). Both genes were also identified by Heshof et al. (2014) and show similar identities to Alox15 (LoxA, 25% identity and LoxB, 23% identity). Within human Alox5, Leu368, 373, 414, 607, and Ile406 are necessary for proper alignment of the AA pentadiene for catalysis (Gilbert et al., 2011). Conservative substitutions for Ile406 are present in both LoxA (I406L) and LoxB (I406V) (Supplementary Figure S2). Alox15 activity is intimately associated with active site size: Phe352, Ile417, Met418, and Ile592 play a major role in oxygenation specificity and substitution of Ile592 with alanine alters positional specificity of the enzyme to resemble that of Alox12 (Borngräber et al., 1999). Amino acid residues important for positional specificity in Alox15 are partially conserved in LoxB, but not LoxA (Supplementary Figure S2) (Sloan and Sigal, 1994; Schwarz et al., 2001). Moreover, a distinguishing characteristic of LoxB is the presence of a predicted signal peptide for LoxB, which is absent from LoxA. An examination of published microarray data and mRNA expression studies identified *loxB* but not *loxA* as an expressed gene (Müller et al., 2012). We confirmed these findings by probing for *loxB* and *loxA* expression when *A. fumigatus* was inoculated in different media (GMM or RPMI) and at varying spore concentrations (10^5^–10^6^ spores/mL). After 48 h of growth at 37°C, we observed expression of *loxB* in all conditions tested, but never expression of *loxA* (**Figure 1A**). Thus, due to lack of expression of *loxA*, and the possibility of secretion of LoxB, we focused on this latter gene and created both deletion and overexpression mutants of *loxB* (Supplementary Figure S1).

To verify correct overexpression of the complete LoxB ORF, we assessed levels of 13-hydroxyoctadecadienoic acid (13-HODE) in culture supernatant. 13-HODE has already been documented as the oxylipin product of *A. fumigatus* LoxB and is derived from LA (Heshof et al., 2014). Quantities of 13-HODE observed in supernatants derived from the WT and Δ*loxB* were equivalent to levels observed in our blank negative control, which we attributed to signal noise in the total ion chromatogram (**Figures 1B**). Furthermore, we observed only marginal expression of *loxB* in the wild type under the conditions tested (**Figure 1A**). However, a significant increase in 13-HODE levels was observed in the strain overexpressing full-length *loxB* (**Figure 1B**).

### *Aspergillus fumigatus loxB* Affects Programmed Germination

A previous study had shown changes in development of *A. fumigatus* exposed to disks soaked in AA (Tsitsigiannis et al., 2005), thus we investigated this response microscopically to determine if *loxB* was important for this alteration in development. Both AA and LA were tested at 2.0, 1.0, and 0.5 μg/μL in GMM + 0.5% tergitol (to aid in PUFA solubility) with ethanol used as a vehicle. The degree of germination was quantified by assessing the percentage of DS that had progressed to the SS or GL stage at a particular timepoint.

Tergitol was verified to have no effect on SS or GL formation compared to GMM media (Supplementary Figure S3). At 2.0 and 1.0 μg/μL of AA or LA, all spores remained arrested at the DS stage: even after 24 h at 37°C, no SS were observed (data not shown). At 0.5 μg/μL AA, after 15 h at 37°C, the percentage of wild type spores progressing to SS and GL was 30% less than spores grown in the mock condition (**Figure 1C**). However at 0.5 μg/μL LA, no significant difference in the total number of SS and GL was recorded in wild type (**Figure 1D**). We were curious whether the presence of these PUFAs had an effect on *loxB* expression, so quantitative RT-PCR was done from the wild type strain grown in the presence of vehicle (ethanol) and 0.5 μg/μL AA and LA for 29 h at 37°C. We found that AA increased *loxB* expression two-fold, whereas LA increased *loxB* expression, but not significantly from the vehicle treatment (**Figure 1E**).

Based on the differences in growth observed at 0.5 μg/μL AA and that *loxB* expression was induced two-fold in the presence of AA, we quantified the differences in SS and GL levels upon *loxB* deletion (*ΔloxB*) or overexpression (*OE::loxB*). Timecourse microscopy was used to monitor SS and GL formation for the wild type and *loxB* mutants over a period of 36 h under mock control and 0.5 μg/μL AA treatments. In the control treatment, the *OE::loxB* strain consistently had more spores germinate through 15 h post inoculation (hpi) whereas the *ΔloxB* and *ΔloxB* complemented [*loxB(c)*] strains had equivalent numbers of SS and GL as wild type through 15 hpi (**Figure 1F**). In the AA treatment, the *OE::loxB* strain again transitioned to SS and GL stages faster than the other three strains. However, in contrast to the control treatment, the proportion of *ΔloxB* DSs that became SS or GL was less than wild type and complement at all time points tested in AA (**Figure 1F**). Due to the fact that differences in all treatments could be observed at the 15 hpi, all further experimentation were quantified at this time point unless otherwise specified.

Next we asked if the delay and acceleration in germination of *ΔloxB* and the *OE::loxB* strains, respectively, was a function of AA treatment only. Therefore, we compared germination of the three strains in 0.5 μg/μL LA to the wild type after 15 hpi grown in the same condition. As shown in **Figure 1G**, whereas the *OE::loxB* strain again showed accelerated germination processes, the *ΔloxB* presented the same phenotype as the wild type and complemented strains. Thus the *ΔloxB* delay in SS and GL formation was a function of AA, whereas accelerated germination of the *OE::loxB* strain was not.

To confirm that *OE::loxB* increased the transition of DS to SS and GL stages and was not an artifact from strain development or background, additional *OE::loxB* strains were examined in two different backgrounds; including strains containing both an *OE::loxB* and wild type *loxB* allele (TGJF 1.5 and TGJF1.7) or just *OE::loxB* allele in a *loxB* deletion background (TGJF34.9 and TGJF34.10). All strains had a higher percentage of SS and GL than wild type on GMM + 0.5% tergitol, regardless of background (Supplementary Figure S4A).

Finally, we asked if AA had any differential impact on the *loxB* mutants and wild type in post-germination growth (mycelium). An established mycelium was developed by growth of the various strains overnight followed by a baseline mycelium reading. No inherent difference in growth were observed among the strains before AA treatment (Supplementary Figure S4B). AA was then added at the specified concentrations to the various strains. In contrast to the *loxB-*dependent regulation of germination by AA, while AA did generally inhibit mycelial growth in a dose-dependent fashion (the OD_600_ actually decreased in some treatments), there was no difference in growth among the *loxB* strains on AA amended medium (Supplementary Figure S4C).

### LoxB Loss Delays Entry into Swollen Spore Stage Whereas Overexpression of *loxB* Accelerates Entry into the Swollen Spore Stage

**FIGURE 2:**
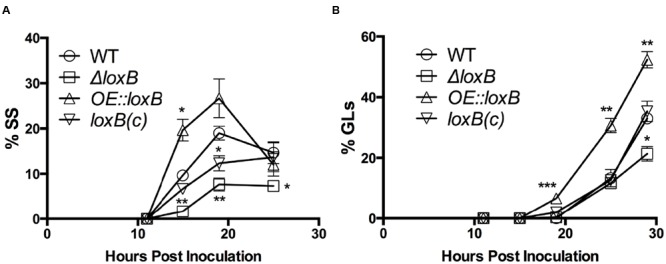
**LoxB affects SS and germling formation.** Breakdown of the total number of germinating spores by SS **(A)** or germlings (GLs) **(B)** in the presence of AA. Overexpression of *loxB* yields a higher number of GLs through 29 hpi. Difference in the number of SS were more significant at multiple timepoints, with the greatest difference among the strains observed 15 hpi. Values represent average of *n* = 3 trials ± SEM and Student’s *t*-test was used to identify statistical differences, ^∗^*p* < 0.05, ^∗∗^*p* < 0.01, ^∗∗∗^*p* < 0.001.

To clarify if *loxB* overexpression or loss impacted specific germination stage transitions on AA, SS and GL were individually assessed. Differences in the number of SS were observed at several time points. Particularly at 15 and 19 hpi, the *ΔloxB* strain consistently had less SS than wild type or the *loxB(c)* strains. The *OE::loxB* strain maintained a higher number of SS that peaked approximately 19 hpi (**Figure 2A**). This phenomenon likely accounted for the corresponding increase in GLs, which were higher at all time points in the *OE::loxB* mutant, but did not appear earlier than GL formation in the wild type (**Figure 2B**). No difference in the number of GLs was observed between the wild type, *ΔloxB*, and *loxB(c)* strains until 29 hpi at which the number of *ΔloxB* GLs was significantly less than the wild type or *loxB(c)* strains (**Figure 2B**).

### Disruption of the LoxB Signal Peptide Impairs Germination in Presence of AA and Affects Protein Localization

**FIGURE 3:**
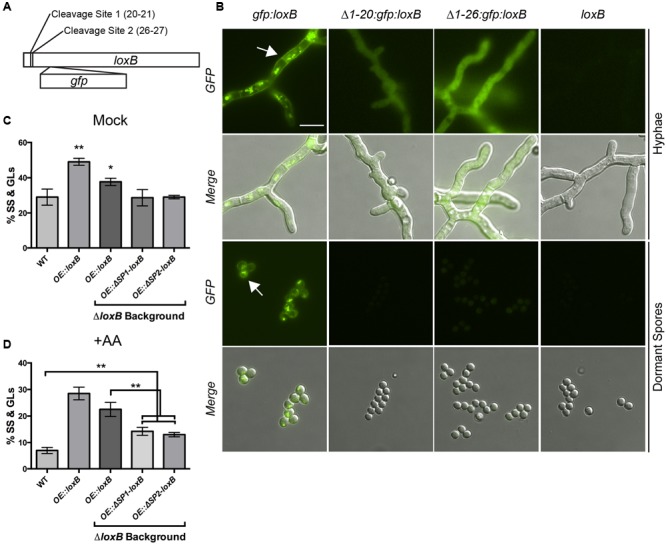
**Disruption of signal peptide affects LoxB localization and dormant spore (DS) maturation. (A)** Location of the putative signal peptide cleavage sites predicted by Signal P 4.0 server (Petersen et al., 2011). Cleavage site are predicted between resides 20 and 21 or residues 26 and 27. **(B)** Localization of various permutations of GFP-LoxB fusion proteins. Full-length GFP-LoxB protein localizes to the cell wall, septa, and puncta within hyphae and cell wall and puncta within DSs. GFP-LoxB protein lacking the first 20 or 26 amino acids localizes throughout the cytoplasm of the hyphae only and is undetectable in DSs. A strain overexpressing untagged full-length *loxB* was used as a fluorescence negative control. **(C)** Spores derived from strains overexpressing truncated versions of *loxB* (*Δ1-20: OE:ΔSP1-loxB, Δ1-26*: *OE:ΔSP2-loxB*) germinate to the same degree as wild type spores, whereas spores derived from strains expressing full-length *loxB* in wild type and *ΔloxB* backgrounds maintain a higher degree of SS and germlings (GLs) than wild type, similar to what was observed in Supplementary Figure S4A. **(D)** Upon incubation with 0.5 μg/μL AA, DSs derived from strains overexpressing truncated versions of *loxB* produce significantly more SS and GLs than wild type. However, the percentage of DSs that proceed to SS or GLs is still significantly less than those expressing full-length versions of *loxB*. Values represent average of *n* = 3 ± SEM and Student’s *t*-test was used to identify statistical differences, ^∗^*p* < 0.05, ^∗∗^*p* < 0.01, ^∗∗∗^*p* < 0.001.

Heshof et al. (2014) commented that fungal LOX with a C-terminal valine also contain an N-terminal signal sequence, suggestive of a protein trafficked through the canonical ER-Golgi apparatus for secretion. LoxB has two putative cleavage sites demarcating the signal peptide region: one between residues 20 and 21 and another between residues 26 and 27 according to predictions by the Signal P 4.0 server (**Figure 3A**) (Petersen et al., 2011).

To visually investigate LoxB localization, signal peptide deficient and full-length versions of LoxB were tagged with GFP and transformed into *A. fumigatus* (see Materials and Methods, Supplementary Figures S1A,D). Full-length GFP:LoxB localized to punctate spots within hyphae but also the cell wall and septa, as is typical of secreted proteins (**Figure 3B**, white arrow) (Khalaj et al., 2001; Lim et al., 2014). In spores, full-length GFP:LoxB localized on the periphery of DS (**Figure 3B**, white arrow) as well as in puncta within the spore. When the first 20 N-terminal residues were absent, GFP:LoxB protein was found uniformly throughout the cytoplasm of hyphae with no localization to the cell wall or septa, and at undetectable levels in DS. The same cytoplasmic localization was observed when the first 26 N-terminal residues were deleted, and barely detectable fluorescent signal in DS. The strain overexpressing untagged full-length LoxB was used as a negative fluorescence control for both hyphae and spores (**Figure 3B**).

To determine if the LoxB signal peptide was important for germination, we created two overexpression constructs of *loxB* lacking the signal peptides. Strains overexpressing versions of *loxB* lacking the first 20 N-terminal residues (*OE::ΔSP1-loxB)* or the first 26 N-terminal residues (*OE::ΔSP2-loxB*) were transformed into a *ΔloxB* background (Supplementary Figures S1A,D) as the same accelerated germination phenotype was observed in this background as in the wild type (Supplementary Figure S4A). In control GMM +0.5% tergitol media (mock), *OE::ΔSP1-loxB* and *OE::ΔSP2-loxB* strains germinated the same as wild type in contrast to the faster germination of the two *OE::loxB* strains in different backgrounds (**Figure 3C**).

We also noted that the number of functional *loxB* alleles impacted germination as the strain with both a wild type and *OE::loxB* allele showed accelerated germination over the strain with only an *OE::loxB* allele (^∗∗^*p* < 0.01 vs. ^∗^*p* < 0.05, **Figure 3C**). Upon addition of 0.5 μg/μL AA, strains overexpressing versions of *loxB* lacking signal peptides had significantly less SS and GLs than strains overexpressing full-length *loxB*. However, all of these strains increased progression to SS and GLs compared to the wild type (**Figure 3D**), suggestive of partial activity of lipoxygenase in these strains despite mislocalization of LoxB.

### The Alox5 Metabolite 5-HETE Promotes Swollen Spore and Germling Formation in the Presence of AA

The differences in germination stage progression of both *OE::loxB* and *ΔloxB* strains treated with AA (**Figures 1–3**) suggested a possible role for an AA LoxB generated oxylipin in *A. fumigatus* germination. We hypothesized that the delay of DS transition to SS and/or GLs in the *ΔloxB* strain in AA medium was due to either AA toxicity (which could be remediated by AA oxygenation) or lack of an induction cue, possibly from an oxylipin derived from AA. Given the similarity of LoxB to both human Alox5 and Alox15, we attempted to investigate whether *loxB* produced the AA products 5-HETE or 15-HETE, the stable surrogates for 5- or 15-Hydroperoxyeicosatetraenoic acid (5-HPETE, 15-HPETE) of the respective pathway in mammals, but were unsuccessful given our current reagents and technology.

**FIGURE 4:**
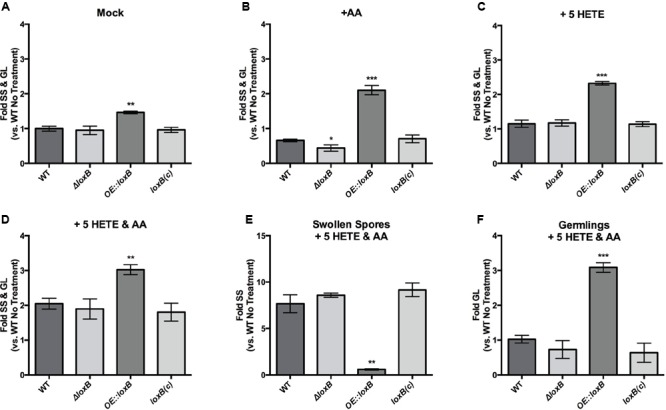
**5-HETE promotes SS and germling formation.** Quantification of SS and germlings (GL) for *loxB* strains grown in GMM +0.5% tergitol (mock) **(A)**, 0.5 μg/μL AA **(B)** or 5-HETE **(C)** or both **(D)**. While exposure to AA reduces the number of SS and GLs compared to wild type in the *ΔloxB* strain, exposure to 0.5 μg/μL 5-HETE, or AA and 5-HETE maintains or increases the proportion of SS and GLs compared to the mock treatment, respectively. **(E)** SS are significantly increased compared to the mock treatment in the presence of AA and 5-HETE. Few SS were observed in the *OE::loxB* strain since all DSs had already progressed to the GL stage **(F)**. Values represent average of *n* = 3 ± SEM and Student’s *t*-test was used to identify statistical differences, ^∗^*p* < 0.05, ^∗∗^*p* < 0.01, ^∗∗∗^*p* < 0.001.

Despite our inability to determine if LoxB produced HETEs, we proceeded to examined if 5-HETE (the product of human Alox5 and implicated in asthma, Hallstrand and Henderson, 2010) could alter the germination dynamics of *A. fumigatus.* All four strains (*ΔloxB, OE::loxB*, wild type and complemented *ΔloxB*) were grown on mock, 0.5 μg/μL AA alone, 0.5 μg/μL 5-HETE alone, or both metabolites at 0.5 μg/μL. Germination profiles were repeated as before on mock and AA medium (**Figures 4A,B**) with *ΔloxB* delayed on AA (**Figure 4B**). Treatment with 5-HETE resulted in a profile similar to that of control medium where *ΔloxB* had equivalent levels of SS and GLs as the wild type and complement while *OE::loxB* showed accelerated germination (**Figure 4C**). This restoration of germination levels of *ΔloxB* to wild type levels by 5-HETE still did not distinguish between inhibition by AA or induction by 5-HETE. Thus, we assessed germination under simultaneous treatment with both compounds at the same concentration. Germination rates increased for all four strains, with *OE::loxB* still germinating faster than the other three strains (**Figure 4D**).

**FIGURE 5:**
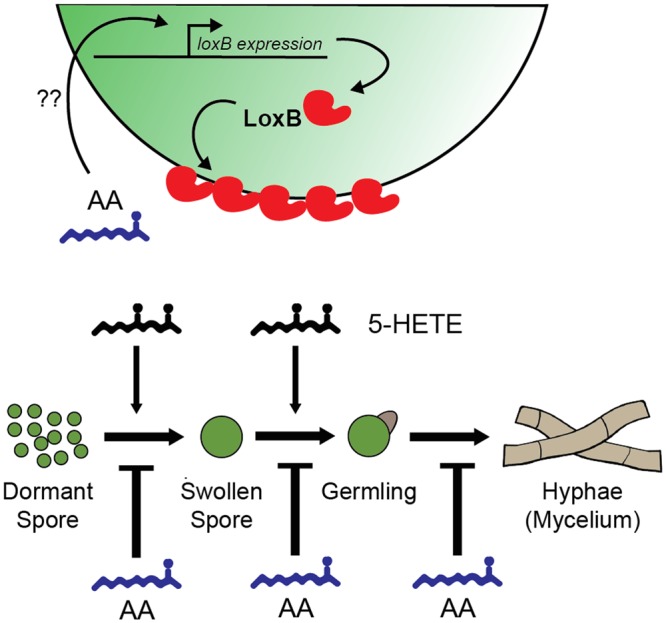
**Hypothetical model for the effects of arachidonic acid (AA), LoxB, and oxylipins on the germination of spores in *A. fumigatus.*** AA has inhibitory effects on each stage of *A. fumigatus* development from DSs to mycelial network. The inhibitory effects of AA can be suppressed in a *loxB-*dependent fashion through the AA-dependent up-regulation of *loxB* via unknown mechanisms. Upon proper localization, LoxB may convert the germination program inhibitor AA to stimulatory 5-HETE, creating a synergistic environment that further accelerates germination.

Because the *ΔloxB* strain showed similar germination rates as the wild type and complement control, it appeared that 5-HETE could act as a germination cue. To further tease apart the effects of the AA/5-HETE co-treatment and to understand the increases in germination for all strains, we counted just SS (**Figure 4E**) or GLs (**Figure 4F**). Progression to SS was greatly increased in wild type, *ΔloxB* and complement (**Figure 4E**), whereas *OE::loxB* had largely exited out of this stage and was primarily in the GL stage (**Figure 4F**). Together, this data allowed us to propose a model of inhibition of germination by AA countered by stimulation by the AA derived oxylipin 5-HETE that resulted in an apparent synergistic effect on germination (**Figure 5**).

## Discussion

Programmed spore germination is a critical process for survival and entry into vegetative growth for all fungi. The spore should remain dormant in unfavorable environments and respond to cues in favorable settings for entry into vegetative growth. Despite the importance of germination, surprisingly little is known about this process, particularly for human pathogenic fungi. Here we present unexpected insight into this programmed event in *A. fumigatus* where we find an important role for a lipoxygenase in accelerating the transitions of DS to SS and GL stages of germination. We find both the substrate and product of human Alox5, AA and 5-HETE respectively, can act as key molecules in these transitions, where 5-HETE appears to act as a germination accelerator for this species.

To our knowledge, our work is the first to identify molecules directly affecting the germination program in *A. fumigatus*. Thus far, deletion of the G protein RasA or spermidine synthase SpdA have been shown to impact the SS to GL stage in *A. nidulans* (Som and Kolaparthi, 1994; Jin et al., 2002) and inactivation of the RasA homolog in *A. fumigatus* also delays germination, but detailed assessment was not analyzed in this mutant (Fortwendel et al., 2008). However, loss of function RasA or SpdA mutations have pleiotropic effects on fungal growth resulting in significantly aberrant morphologies beyond delays in spore germination. In contrast, both deletion and over-expression of *loxB* appear to only affect the transition of germination stages with the former mutant (*ΔloxB*) only showing a phenotype when AA is incorporated into the media.

Several PUFAS have been shown to be important in the germination process of multiple fungi, often as an inhibitor (Liu et al., 2008). However, AA has not been investigated except as a potential germination inducer in *Mortierella alpine* (Lounds et al., 2007). This fungus, which produces high levels of endogenous AA, displays a similar germination program as *A. fumigatus* with rapid AA depletion during the SS stage for this species. The authors speculated that this was related to fatty acid desaturase activity but no mechanism was described. Here we suggest a process in *A. fumigatus* where *loxB* is induced by AA, the enzyme is then secreted and exogenous AA is potentially converted to unknown oxygenated compounds, one of which potentially being 5-HETE, which acts as a germination cue in the presence of AA (**Figure 5**).

Both AA and 5-HETE altered the dynamics of the germination program in a *loxB-*dependent fashion. Whereas AA treatment delays germination in all strains, overexpression of *loxB* overcomes AA suppression at the DS to SS stage. In contrast, *ΔloxB* is delayed at this same transition; this delay may be associated not with AA inhibition *per se* but with the lack of an induction cue (such as 5-HETE), as *ΔloxB* presents an identical phenotype as wild type in both 5-HETE and 5-HETE/AA treatments (**Figures 4C,D**). We recognize an apparent synergistic effect on germination when both AA and 5-HETE are used to simultaneously treat spores: in the presence of 5-HETE, the *ΔloxB* strains germinates to the same degree as the WT, but with simultaneous treatment has germination two-fold greater than individual treatment. This is an interesting phenomenon that we feel should be tested with a greater repertoire of oxylipin compounds in further studies. This phenomenon appears specific to AA, as LA did not affect germination in either a *loxB-*dependent or independent manner. We suggest that possession of a lipoxygenase presents an advantage to *A. fumigatus* in PUFA rich environments (specifically those with AA) through acceleration of the germination program.

As hypothesized in an earlier study (Heshof et al., 2014), LoxB is a secreted protein and its proper localization is required for full function in germination dynamics (**Figures 3C,D**). GFP-tagged versions of full-length LoxB revealed the protein localized to the cell wall and septa of the mycelium as well as the cell wall of DS when overexpressed (**Figure 3B**), which is the expected localization pattern for secreted fungal proteins such as the fumiquinozoline C oxidoreductase, *fmqD* in *A. fumigatus* (Lim et al., 2014) and a glucoamylase-GFP fusion protein in *A. niger* (Khalaj et al., 2001). Truncated GFP-LoxB no longer localized to the cell wall or septa but appeared diffused throughout the cytoplasm of the cell, similar to localization of truncated versions of FmqD-GFP lacking the signal peptide sequence carried out by Lim et al. (2014). Secretion of LoxB would aid in rapid conversion of environmental AA (a germination inhibitor) to germination stimulators such as 5-HETE. However, whether *A. fumigatus* is able to synthesize 5-HETE from AA remains to be determined.

We found 5-HETE increased the proportion of SS in a *loxB-*independent manner. The proportion of GLs was significantly greater in all strains when compared to AA treatment, suggesting that 5-HETE promotes the formation of SS and GLs (**Figure 5**). Although we only assessed the impact of 5-HETE on germination (due to cost of these metabolites), it is possible that other induction cues, including additional oxylipins, may affect germination. Oxylipins thus far have been described as inhibitors of germination. In some studies, the volatile 1-octene-3-ol was found to inhibit germination in a density dependent manner (Fischer et al., 1999; Chitarra et al., 2004; Herrero-Garcia et al., 2011), although not in *A. flavus* (Miyamoto et al., 2014). Also, several plant-derived oxylipins have inhibitory properties against spore germination of the oomycetes *Phytophthora infestans* and *P. parasitica*, and the ascomycete *Botrytis cinerea* (Prost et al., 2005). To the best of our knowledge this is the first example of an oxylipin inducer of fungal spore germination.

The transition from DS to SS and eventual GLs is a critical fungal process for host recognition and establishment of infection of *Aspergillus fumigatus.* This opportunistic human pathogen causes diseases ranging from invasive aspergillosis (IA) in hypo-immune states or allergic bronchopulmonary aspergillosis (ABPA) in hyper-immune states. Host recognition of *A. fumigatus* spores is initially delayed upon inhalation because the hydrophobic rodlet layer covering the resting spore evades immune recognition (Aimanianda et al., 2009). As the spore swells, the proteinaceous rodlet layer is shed, exposing numerous cell wall components that function as pathogen-associated molecular patterns (PAMPs) (for review, see van de Veerdonk et al., 2008). In ABPA, numerous PAMPs on SS can elicit hypersensitivity responses and as extensive hyphal growth is not associated with this disease, it is presumed fungal development is largely limited to SS and/or GL stages. In contrast, in IA, the host is unable to destroy SS or GLs and the fungus transitions to invasive hyphal growth.

This work may support a role of LoxB in potentiating disease associations at two levels, one by altering host recognition of the spore and one where LoxB metabolites – identical to several immunomodulatory oxylipins produced by the host itself (e.g., 13-HODE) – could contribute to harmful inflammatory processes. Considering the first point, it is known that germination kinetics have a profound impact on host recognition and clearance of fungal spores. The speed at which host immune cells can clear fungal spores is dependent on the size and shape of the spore and interactions with spore surface receptors (PAMPs) (Hoffmann et al., 2010). PAMPs are masked by a layer of rodlet proteins on DSs of *A. fumigatus*, which are shed during isotrophic growth to the SS stage. Many studies have identified *A. fumigatus* PAMPs that influence macrophage clearance of *A. fumigatus* spores, such as ß-1,3-glucan (Hohl et al., 2005; Luther et al., 2007), mannans (Willment and Brown, 2008), and lectins (Kerr et al., 2016). Once phagocytosed, reactive oxygen killing through macrophage NADPH oxidase (Philippe et al., 2003) and acidification of the phagolysosome (Ibrahim-Granet et al., 2003) are thought to be the predominant ways macrophages neutralize spores. The *A. fumigatus* spore pigment DHN melanin suppresses acidification (Jahn et al., 2002) and is thought to allow for rapid germination and escape from the macrophage, but also renders *A. fumigatus* more vulnerable to antifungal agents, since hyphae are generally more susceptible than spores (Diamond, 1988; Slesiona et al., 2012). In changing germination kinetics, LoxB activity – or lack of it – could have influence on all of these fungal developmental factors and subsequent host/microbe interactions.

Secondly, LoxB metabolites (such as 13-HODE and potentially 5- and 15-HETE) could exacerbate inflammatory responses in the host. Examples of oxylipins influencing immune and airway dynamics are numerous. 20-HETE and 13-HODE are both implicated in airway hyperresponsiveness (Henricks et al., 1995; Cooper et al., 2010), while 13-HODE has also been implicated in disruption of airway epithelial cells calcium homoeostasis and mitochondrial structure, resulting in bronchial cell injury (Mabalirajan et al., 2013). This injury leads to severe airway remodeling, increased airway neutrophilia, and an increase in stress-related pro-inflammatory cytokine release.

## Conclusion

We have identified an enzyme, LoxB, which could provide *A. fumigatus* a competitive edge in colony establishment in varied environments, particularly those where AA is present. The mammalian host is just one such environment where kinetics of spore germination are intimately linked with immune recognition and clearance. Considering that germination is the first developmental program of fungal growth where antimicrobial intervention could reduce the incidence of infection, our findings also provide insights into *A. fumigatus* germination processes which may be useful for therapeutical studies. Whether the accelerated germination phenomenon observed in the *OE::loxB* strain impacts host recognition and invasive growth is the focus of future work. If the germination process is mediated through oxylipin production, several Lox inhibitors have been developed for treatment of asthma and other inflammatory diseases (Eleftheriadis et al., 2015; Suh et al., 2015), and it would be interesting if measures along these lines could impact diseases caused by *A. fumigatus.*

## Author Contributions

GF and NK conceived experiments and wrote manuscript. WB assisted GF with experimentation. JP and TD assisted in strain development. JY and BH carried out experimentation.

## Conflict of Interest Statement

The authors declare that the research was conducted in the absence of any commercial or financial relationships that could be construed as a potential conflict of interest.

## Footnotes

1 http://www.aspergillusgenome.org

2 http://www.graphpad.com
