# Supplementary material for: Lipoxygenase Activity Accelerates Programmed Spore Germination in Aspergillus fumigatus
Source: Front Microbiol. 2017 May 9;8:831. doi: 10.3389/fmicb.2017.00831 (PMC5422543; doi:10.3389/fmicb.2017.00831)
Supplement: Supplementary file 1 [file Data_Sheet_1.DOCX]

**Supplemental Information**


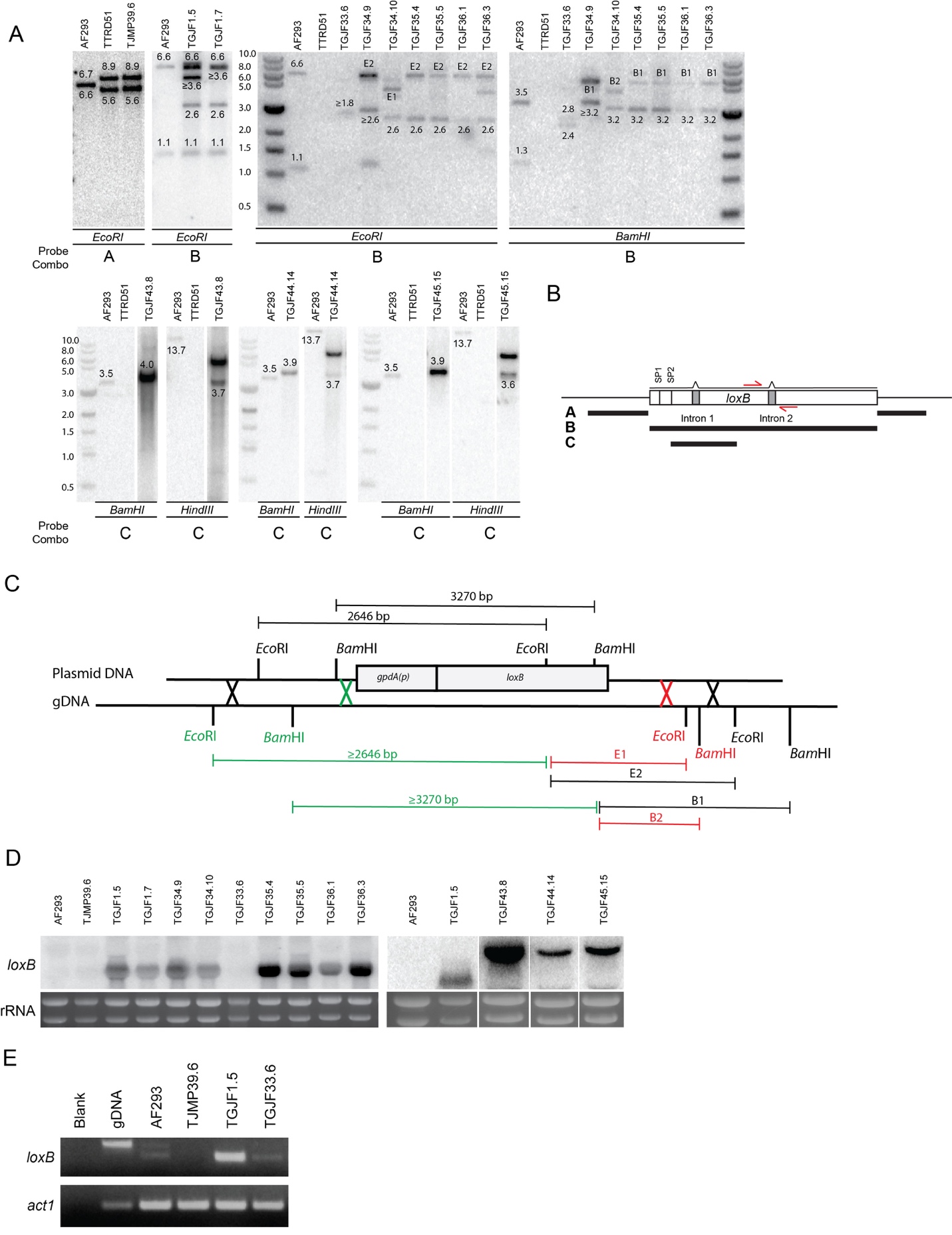


**Figure S1.** Development and verification of *A. fumigatus* *loxB* mutant strains. **(A)** Auxotrophic strains in the AF293 background were utilized to make various *loxB* mutants. As described in Material and Methods, various disruption cassettes and plasmid constructs were used to transform AF293.6 or AF293.1 auxotrophic strains to prototrophy. Verification of the appropriate strain was carried out via Southern blot using the specified restriction enzyme and radiolabeled DNA probe as diagramed in **(B).** TTRD51 (*∆loxB* auxotroph), TJMP39.6 (*∆loxB* prototroph, used in this study), TGJF1.5 & TGJF1.7 (*OE::loxB*, used in this study), TGJF33.6 (*loxB* complement in *∆loxB* background, used in this study), TGJF34.9 & TGJF34.10 (*OE::loxB* in *∆loxB* background, used in this study), TGJF35.4 & TGJF35.5 (*OE::[ΔSP1]:loxB* in *∆loxB* background, used in this study), and TGJF36.1 & TGJF36.3 (*OE::[ΔSP2]:loxB* in *∆loxB* background, used in this study), TGJF43.8 (*OE::gfp:loxB* in *∆loxB* background, used in this study), TGJF44.14 (*OE::[ΔSP1]:gfp:loxB* in *∆loxB* background, used in this study), and TGJF45.15 (*OE::[ΔSP2]:gfp:loxB* in *∆loxB* background, used in this study) were all compared to the AF293 (WT) strain and confirmed. SP1 and SP2 refer to the two putative N-terminal signal peptide regions as diagramed in **(B). (B).** Gene structure for *loxB* and regions used for radiolabeled probes in Southern and northern blots. **(C)** Restriction digest pattern used to confirm ectopic integration of *loxB* constructs. Depending on the location of the recombination event, different size products are identified via Southern blot. **(D)** Northern analysis of total RNA isolated from mycelial tissue. The entire ORF of *loxB* was used to probe for transcript. Ribosomal RNA was used as a loading control. Note the shift in the band size for the gfp-*loxB* fusion construct (TGJF43.8, TGJF44.14, and TGF45.15). **(E**) To further verify deletion and complementation of *loxB,* semi RT-PCR was carried out of cDNA produced from total RNA (see Materials and Methods). Amplicons corresponding to cDNA from spliced *loxB* mRNA (vs. genomic DNA or gDNA) were identified in the wild type (AF293), *loxB* complement (TGJF33.6), and *loxB* overexpression (TGJF1.5) strains. No amplicon was detected in the deletion strain (TJMP39.6). *act1* was used as a cDNA loading control.


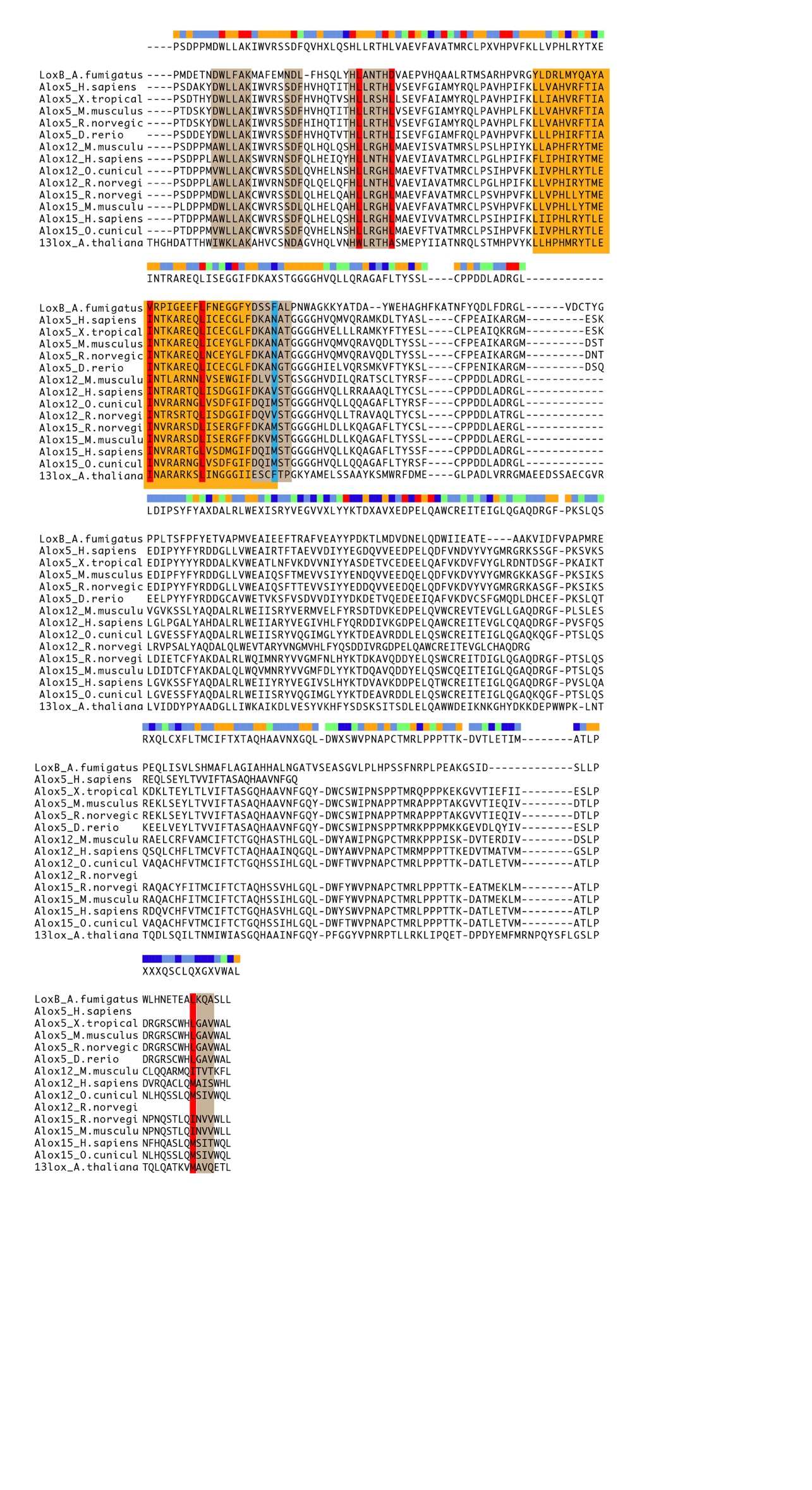


**Figure S2.** Multiple sequence alignment of *A. fumigatus* LoxB with various 5-,12-,and 15-Lox sequences as well as a 13-Lox sequence from *Arabidopsis thaliana* highlighting conserved Lox-specific resides in the various sequences*.* 5-Lox residues important for proper alignment of the arachidonic acid pentadiene motif within the active site are highlighted in red (Gilbert, *et al.,* 2011). Residues important for catalytic activity, metal ion coordination, and sequence determinants for positional specificity of oxygenation are depicted in tan (Schwarz *et al.,* 2001). A residue implicated in positional specificity is shaded in blue (12- vs. 15-Lox) (Sloan & Sigal, 1994).

hwarz


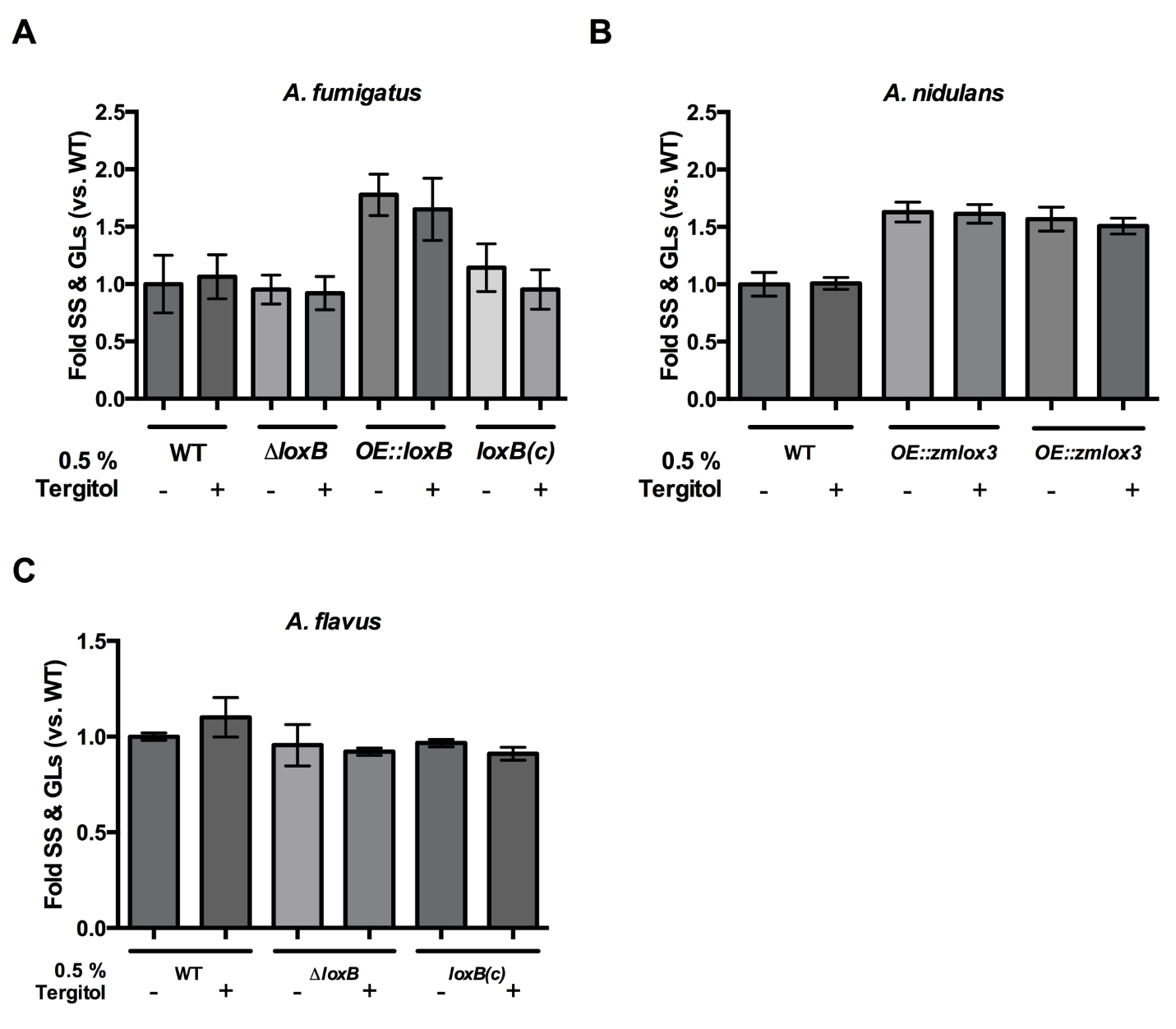


**Figure S3**. Effects of 0.5% tergitol on various lipoxygenase mutants in *A. fumigatus.* Values represent average of n=3 trials ± SEM and Student’s t-test was used to identify statistical differences, *p<0.05, **p<0.01, ***p<0.001.


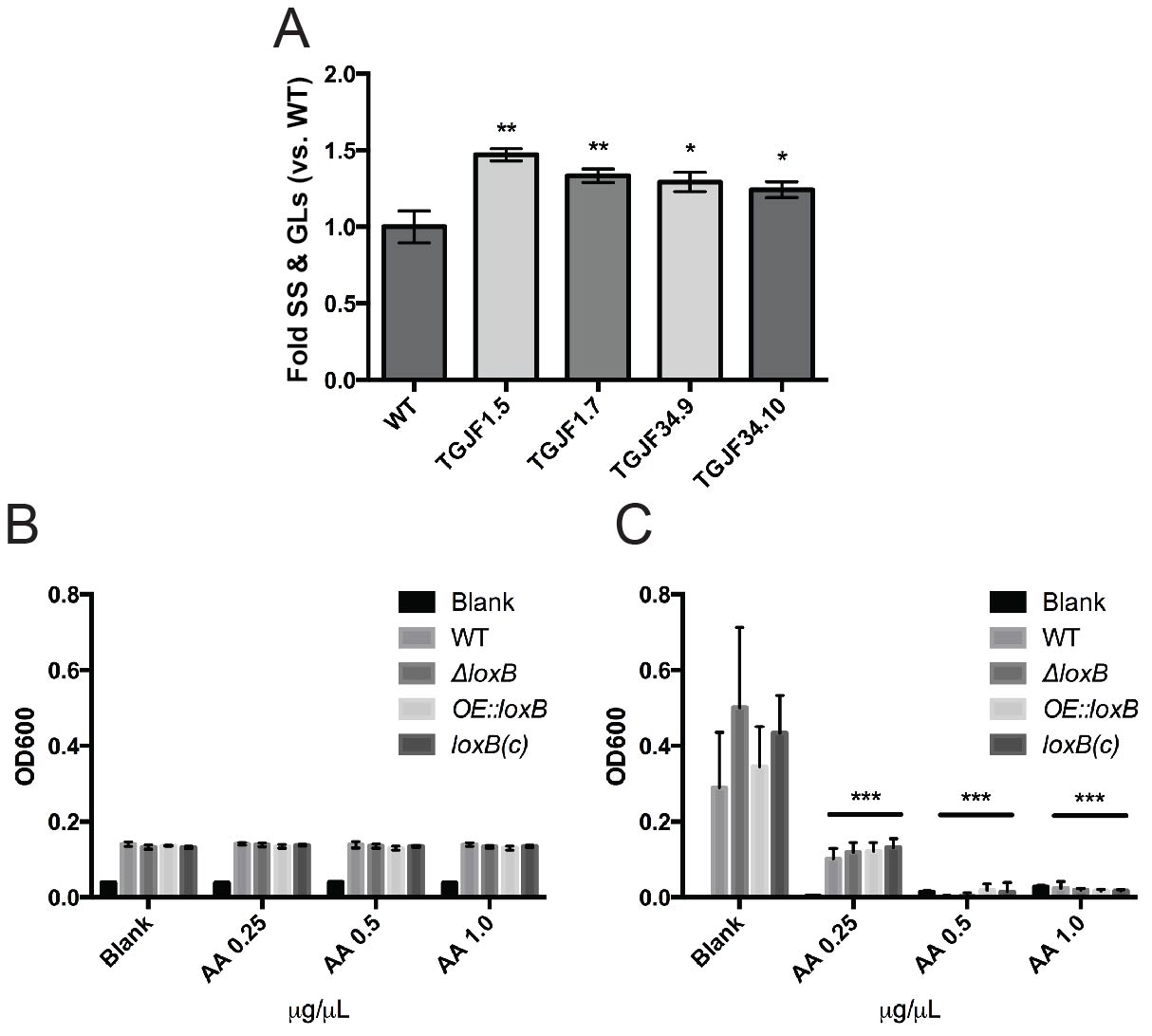


**Figure S4**. Germination and *loxB* expression in various *A. fumigatus loxB* mutants. **(A)** The proportion of swollen spores (SS) and germlings (GL) in *A. fumigatus* mutants overexpressing *loxB* in wild type (TGJF1.5 and TGJF1.7) or *∆loxB* (TGJF34.9 and TGJF34.10) backgrounds is elevated compared to wild type. Mycelial growth of wild type and *loxB* mutant strains. 1 X 10^5^ spores were inoculated in 100 µL of GMM +0.5% tergitol and grown in a 96 well plate overnight, after which and OD_600_ reading revealed no difference in mycelial growth before AA addition **(B)**. Arachidonic acid was then added to the mycelium at a final concentration of 0.25, 0.5, and 1.0 µg/µL and an additional OD_600_ reading collected 22 hours later **(C)**. Arachidonic acid inhibited mycelial growth in a dose-dependent fashion, but *loxB* disruption or overexpression had no differential effect on growth. Values represent average of n=3 trials ± SEM and Student’s t-test was used to identify statistical differences, *p<0.05, **p<0.01, ***p<0.001.

**Table S1. Primers used in this study.**

| **Primer** | **Sequence** | **Purpose** |
| --- | --- | --- |
| *GF gpdA/loxB-loxB(t) F* | 5’-AGCTACCCCGCTTGAGCAGACATCACCATGATGGTCTTCAGTGATTGCCT-3’ | Overexpress *loxB* |
| *GF loxB(t) XbaI Site R* | 5’-GGCGGCCGCTCTAGAAGCAGAC-3’ | Overexpress *loxB* |
| *GF gpdA F* | 5’-CTCTCTACTAGTATCCGGATGTCGAAGGCTTGGGGCACCTGC-3’ | *gpdA(promoter)* |
| *GF gpdA/loxB R* | 5’-CGAGAAAATCAGGCAATCACTGAAGACCATCATGGTGATGTCTGCTCAAG-3’ | *gpdA(promoter)* |
| *JP Afumi argB F* | 5’- GAACGCGGTCTGCATCCAAG-3’ | argB |
| *JP Afumi argB R* | 5’- GAAGGAGAGACCCATACATCC-3’ | argB |
| *TDLoxB P1 F* | 5’-GAAAGACATCCCAGACAA-3’ | 5’ Flank |
| *TDLoxB P2 F* | 5’-GCATGTAAGCACCCTTGTC-3’ | Nested For |
| *TD LoxB P3 R* | 5’-CTTGGATGCAGACCGCGTTCGCCAAAGTGTTTCTCATCGTC-3’ | *loxB* 5’ Flank cassette |
| *TDLoxB P4 F* | 5’-GGATGTATGGGTCTCTCCTTCCGTTTGCTGACGCTGGAAGTA-3’ | *loxB* 3’ Flank cassette |
| *TDLoxB P5 R* | 5’-GTCAGGAAATGGCCTCTGA-3’ | Nested Rev |
| *TDLoxB P6 R* | 5’-ATACCCTGGGCTCGATTAG-3’ | 3’ Flank |
| *GF d1-20:loxB F* | 5’-CCCCGCTTGAGCAGACATCACCATGCTGCCAGTGGTTCCCGGCCAAACAG-3’ | *loxB* Signal Peptide Deletion (1-20) |
| *GF d1-26:loxB R* | 5’-CCCCGCTTGAGCAGACATCACCATGCAAACAGTGATGGAACCTTCCGCAGC-3’ | *loxB* Signal Peptide Deletion (1-26) |
| GF loxB Complement F | 5’-  CTCTCTACTAGTTTGGAGACGAGAAATGTGATCCAAAGG-3’ | *loxB* Complement |
| GF loxB Complement R | 5’-  GCTCAGGCGGCCGCTTGGTTTGCAGCATCGTATCAGG-3’ | *loxB* Complement |
| GF loxB-Nterm GFP F | 5’-CTGCCAGTGGTTCCCGGCCAAACAGTGATGAGTAAAGGAGAAGAACTTTTCACTGG-3’ | *loxB gfp* |
| GF loxB-Nterm GFP R | 5’-CCCGTCATCAGGAAGGGCTGCGGAAGGTTCGGCACCGGCTCCAGCGCCTGCACCAGC-3’ | *loxB gfp* |
| GF loxB qPCR F | 5’-CCATGAGCGCTCGTCATCC-3’ | *loxB* qPCR |
| GF loxB qPCR R | 5’-CGCGGTCAAACAGGTCTTGG-3’ | *loxB* qPCR |
| GF loxA seq 1F | 5’-CGAGCCTTTGGTTCCATCG-3’ | *loxA qPCR* |
| GF loxA Probe R | 5’-GCATCGCTGTACCAATCTGG-3’ | *loxA qPCR* |
| FY act1 RT FOR | 5’-CGGCCGTGATCTGACGGAC-3’ | *Actin* qPCR |
| FY act1 RT REV | 5’-AGCTCTGGGAGGCAGTCTG-3’ | *Actin* qPCR |

**Table S1**. PCR primer names and sequences for strain development. All primers were used as described in Materials and Methods.

**Table S2. LOQ and Linearity Range.**

| **Analyte** | **Limit of quantitation [nM]** | **Limit of quantitation**  **[pg]** | | **Linear range**  **[nM]** |
| --- | --- | --- | --- | --- |
| **13 HODE** | 0.10 | 0.30 | 1−500 | |

**Table S2.** Limit of quantification (LOQ) and linearity range for 13-hydroxyoctadecadienoic acid (13-HODE) identification.
